# Short Review on Porous Metal Membranes—Fabrication, Commercial Products, and Applications

**DOI:** 10.3390/membranes8030083

**Published:** 2018-09-18

**Authors:** Bo Zhu, Mikel Duke, Ludovic F. Dumée, Andrea Merenda, Elise des Ligneris, Lingxue Kong, Peter D. Hodgson, Stephen Gray

**Affiliations:** 1 Institute for Sustainable Industries and Liveable Cities, Victoria University, Werribee Campus, P.O. Box 14428, Melbourne, VIC 8001, Australia; bo.zhu@vu.edu.au (B.Z.); mikel.duke@vu.edu.au (M.D.); 2 Institute for Frontier Materials, Deakin University, Waurn Ponds, Geelong, VIC 3216, Australia; ludovic.dumee@deakin.edu.au (L.F.D.); amerend@deakin.edu.au (A.M.); edeslign@deakin.edu.au (E.d.L.); lingxue.kong@deakin.edu.au (L.K.); peter.hodgson@deakin.edu.au (P.D.H.)

**Keywords:** porous metal membranes, membrane fabrication, water treatment, membrane filtration, membrane bioreactors, photocatalysis, catalysis

## Abstract

Porous metal membranes have recently received increasing attention, and significant progress has been made in their preparation and characterisation. This progress has stimulated research in their applications in a number of key industries including wastewater treatment, dairy processing, wineries, and biofuel purification. This review examines recent significant progress in porous metal membranes including novel fabrication concepts and applications that have been reported in open literature or obtained in our laboratories. The advantages and disadvantages of the different membrane fabrication methods were presented in light of improving the properties of current membrane materials for targeted applications. Sintering of particles is one of the main approaches that has been used for the fabrication of commercial porous metal membranes, and it has great advantages for the fabrication of hollow fibre metal membranes. However, sintering processes usually result in large pores (e.g., >1 µm). So far, porous metal membranes have been mainly used for the filtration of liquids to remove the solid particles. For porous metal membranes to be more widely used across a number of separation applications, particularly for water applications, further work needs to focus on the development of smaller pore (e.g., sub-micron) metal membranes and the significant reduction of capital and maintenance costs.

## 1. Introduction

Membrane-based processes have become increasingly attractive due to their ability to reliably remove contaminants, their affordability, and the increasing prominence of energy and water issues necessitating efficient treatment of waste effluents. The application of membrane-based processes is well established for municipal water treatment, with cost effective polymeric microfiltration (MF)/ultrafiltration (UF) and reverse osmosis (RO)/nanofiltration (NF) membranes available. Municipal water and wastewater applications are characterised by low inorganic particulate loads and organic based water contaminants of low to moderate concentration, enabling efficient processing. However, many emerging membrane applications involve the separation and/or the filtration of higher strength contaminant loads or abrasive particles in waste concentrate streams or slurries, or processing of gas and vapours, and require porous or dense membranes with robust mechanical properties, and high thermal and chemical stability.

Inorganic membranes prepared from materials such as carbon, silica, zeolite, and various oxides (e.g., Al_2_O_3_, TiO_2_, ZrO_2_) and metals (e.g., Pd, Ag, and their alloys, and steel) are very attractive in membrane separation due to their higher chemical, thermal, and mechanical resistances, exceptional separation features, and long operational lifetime [1]. Although inorganic membrane materials cost more than organic polymeric membrane materials, they offer some advantages such as temperature stability, resistance towards solvents, narrow pore size distribution, and the opportunity for more sterilisation options [2]. Therefore, they can be widely used for the chemical and pharmaceutical industry and for many water applications, particularly for high temperature, extreme pH, abrasive environments, and high-pressure processes that preclude the use of existing polymeric membranes. Although important progress has been achieved in the fabrication and use of inorganic membranes over the last two decades, extensive application has not yet been achieved when compared to polymeric membranes that currently dominate the industry. High capital costs and low surface area packing are generally regarded as the core hindrance for inorganic membranes [3].

While there are many widespread suppliers and uses of inorganic membranes made from ceramics, there are limited suppliers and applications of metal membranes despite their unique advantages over all membrane materials, including ceramics. Research on metal membranes is currently being intensified due to their higher mechanical strength, high temperature stability, easier sealing, and integrated processing when compared to their ceramic and polymeric counterparts [4]. Most metal membranes are characterised by a gradient composite structure consisting primarily of a metal base and a metal, metal oxide, or metal alloy separation layer. Metal membranes are generally categorised into dense membranes (such as hydrogen soluble palladium membranes) and porous membranes. This review, however, focuses on porous metal membranes. Porous metal membranes have been studied for numerous applications related to liquid and slurry food preparation and filtration, and for products such as dairy, fruit juice, and alcohol [5,6,7,8]. Metal membranes offer some advantages over the polymeric membranes for these applications because the modules can be cleaned by high pressure back-flushing, thus reducing the use and influence of cleaning chemicals [9]. Another advantage of using metal membranes in food processing is their stability during steam sterilisation [9]. Porous metal membranes have also been applied for water applications such as MF, membrane reactors and bio-reactors, electrolysers, and membrane evaporators [10,11,12,13,14,15,16,17,18,19,20,21]. However, no review has been carried out on the status of application of porous metal membranes. This work aims to highlight the various fabrication techniques that have been used to successfully fabricate porous metal membranes and to critically review the applications of porous metal membranes. Novel fabrication techniques that were recently investigated for potentially producing nanoscale porous metal membranes and future research directions will also be discussed.

## 2. Fabrication of Porous Metal Membranes

Porous metal membranes can be generally classified into two main types: unsupported metal membranes (metal membrane filters) and porous metal supported membranes. Unsupported metal membranes are made from pure or alloyed metals, while supported metal membranes utilise porous metals for the primary structure, and metal, metal oxide, or alloys are used as the membrane selective layer. The preparation of porous metal membranes mainly utilises the techniques used for fabrication of ceramic membranes and dense metal membranes. The morphology of porous metal membranes such as pore size, pore depth and porosity strongly depend on the fabrication technique used for their porous metal frameworks. There is a range of approaches for preparation of porous metal frameworks [9]. Table 1 summaries a number of processes that are used for the fabrication of porous metal frameworks with an achievable pore size range, and their advantages and disadvantages.

Although a range of techniques as listed above can be potentially used for the fabrication of porous metal membranes, one of the main approaches that has been used for fabrication of unsupported porous metal membranes is particle sintering. Sintering of particles is derived from traditional powder metallurgy technology, which involves the hot-compression of micron-sized metal particles or fibres at the softening temperature of the metal to produce a semi-porous network. The major principle of sintering is to bring the particles together forming necks arising from slow coalescence of the soften metals at high temperatures [72]. Stainless steel (SS) is the most commonly used for producing porous metal membranes by particle sintering. Other metals such as gold, silver, copper, nickel, aluminum, magnesium, titanium, chromium, tungsten, and molybdenum can also be used [4]. A range of conditions (e.g., particle size, sintering atmosphere) has a significant impact on the products from the sintering process [4]. Larger particles not only limit neck formation due to the slow rate of mass transport, but also lead to a mechanically weak membrane due to the favourable formation of finger-like macrovoids [73]. The properties of the metal powders (e.g., size, compressibility and reactivity) used for sintering must be well known to determine the most appropriate heating technique and avoid densification by over-heating [74,75]. The pore size formed using this technique is controlled by the average particle deformation induced by the process and the remaining distance between the particles after sintering [9]. Changing the sintering atmosphere in the sintering process can also result in different morphological features [73]. This technique typically leads to large pores (e.g., >1 µm) [27,28,29,30,31,32,76]. Sintering process has been widely used to produce commercial metal filters/membranes [27,28,29,30,31,32,76]. For example, Porvair Filtration manufactures porous SS membrane filters (Sinterflo^®^) from sinter bonded metal powders [29]. Pall Corporation’s AccuSep™ sintered SS filters have a removal rating of 2–5 µm [31], and PMM^®^ metal membrane filters with 2 µm pores [32]. Most recently, a rolling-sintering process was also reported by Park and co-workers [77] to fabricate porous metal membranes with a pore size of ~4.5 µm.

**Figure 1:**
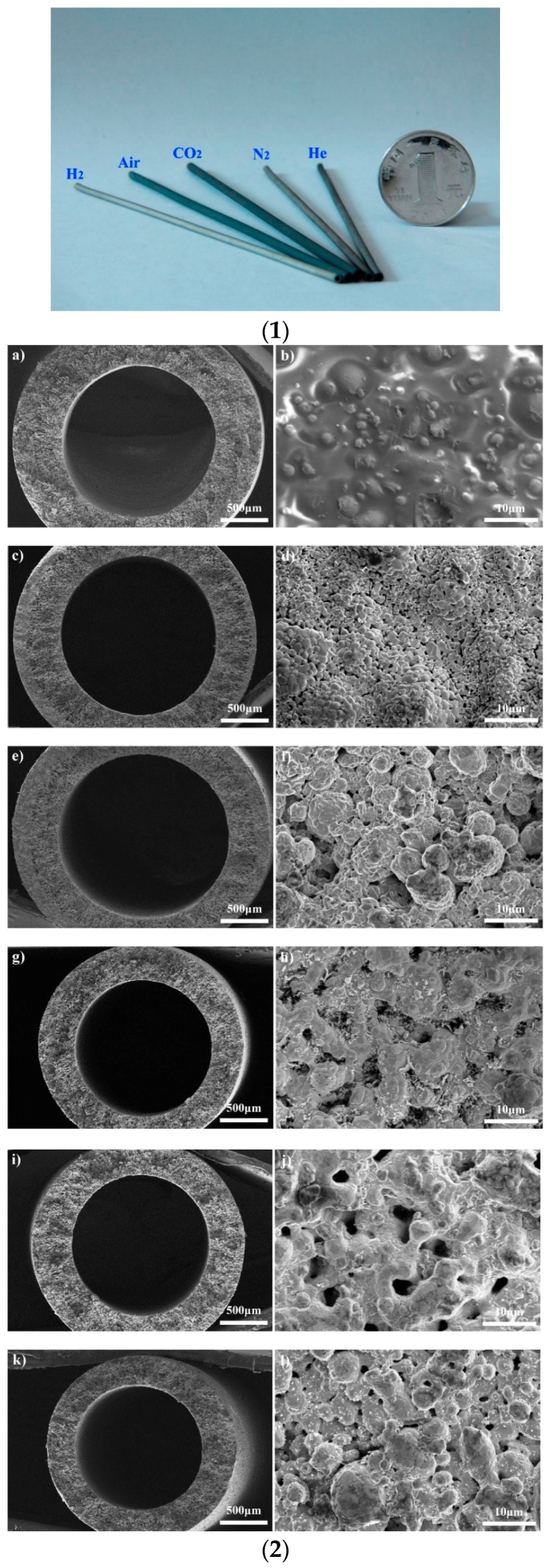
(**1**) Stainless steel hollow fibres prepared under various atmospheres; (**2**) Scanning electron microscopy (SEM) measurements on the cross-sections and surfaces of samples before and after sintering under various atmospheres: fibre precursor (**a**,**b**), with air (**c**,**d**), with CO_2_ (**e**,**f**), with N_2_ (**g**,**h**), with He (**i**,**j**), and with H_2_ (**k**,**l**). Reprinted from “Effects of sintering atmospheres on properties of stainless steel porous hollow fiber membranes” [84], © 2015, with permission from Elsevier.

**Figure 2:**
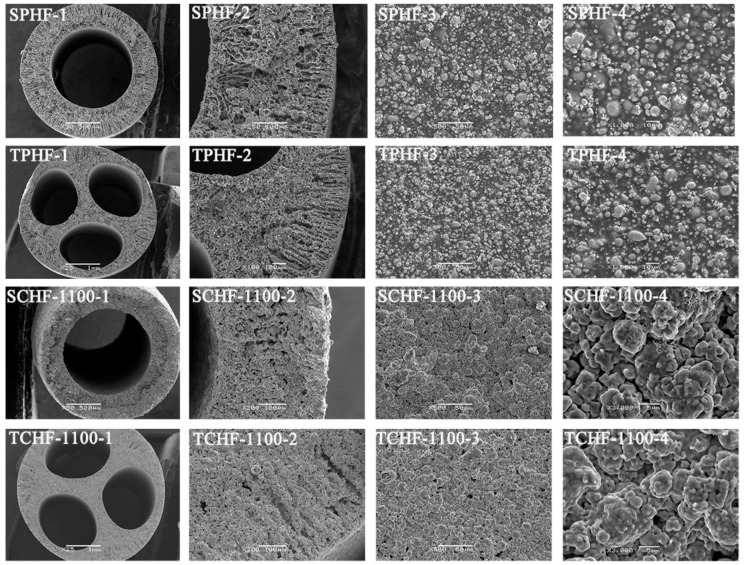
SEM images of the single-channel precursor hollow fibres (SPHF), three-channel precursor hollow fibres (TPHF), single-channel hollow fibres (SCHF), and three-channel hollow fibres (TCHF). 1, full views; 2, enlarged cross-sections (200×); 3, outer surfaces (500×); 4, outer surfaces (1000 and 3000×). Reprinted from “Fabrication, characterization and separation properties of three-channel stainless steel hollow fiber membrane” [83], © 2016, with permission from Elsevier.

**Figure 3:**
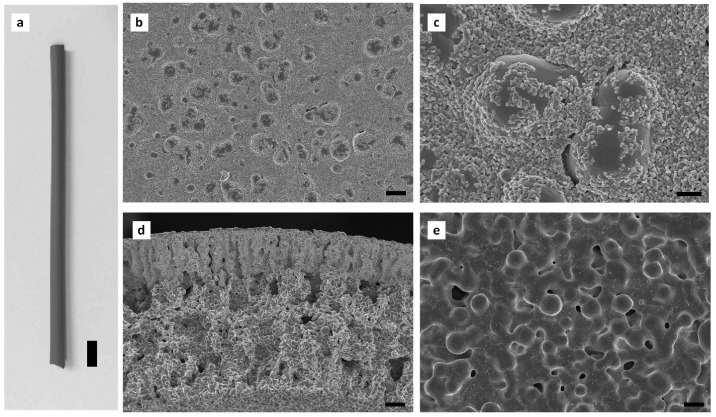
(**a**) Photo of a dual-layer stainless steel (SS)/SS–yttria-stablilised ZrO_2_ (YSZ) hollow fibre. SEM measurements for the SS/SS-YSZ hollow fibres: (**b**,**c**) outer surface (**d**) cross-section, and (**e**) inner surface. (Scale bars: (**a**) 5 mm, (**b**) 4 μm, (**c**) 1 μm, (**d**) 20 μm, (**e**) 4 μm). Reprinted from “High performance stainless steel-ceramic composite hollow fibres for microfiltration” [89].

The sintering technique has great potential to fabricate hollow fibre metal membranes [73,78]. The first metallic hollow fibre membrane was fabricated by Liu et al. [79] by extruding a polymer solution with suspended Ni particles to green hollow fibres, followed by sintering at elevated temperature under an argon atmosphere. Several research groups [73,78,80,81,82,83,84] have recently fabricated SS hollow fibres by micron-sized SS particles. SS hollow fibres can be fabricated by extruding a suspension of SS powders in a polymer solution through a spinneret, followed by coagulating in a phase-inversion process. Once the phase-inversion process is complete, the hollow fibre precursors are sintered at a high temperature. By combining the phase-inversion process with the sintering technique, large amounts of hollow fibres can be continuously produced in one step, and the microstructure of the membranes can be adjusted for targeted applications. Some studies [73,83,84] have been carried out to investigate the effects of sintering conditions on the properties of SS hollow fibres. Schmeda-Lopeza et al. [73] reported that during stainless steel hollow fibre membrane preparation by the sintering process, sintering of fibres in a nitrogen atmosphere resulted in large pores >200 μm, and small pores of 3–5 μm and 0.01–0.1 μm, whilst hollow fibres prepared in an argon atmosphere showed distinct pores in the range of 50–70 μm. Sintering in an argon atmosphere led to the development of some large pores that propagated cracks resulting in lower flexural strain. Sintering with inert gases led to the mass transfer of residual carbon from the binder to the stainless steel powders, resulting in the formation of carbide-rich regions. The sample sintered in argon contained more carbide-rich regions (by area) than the hollow fibres prepared under nitrogen. However, the chemical changes mentioned here did not have an impact on the mechanical properties of the final materials [73]. Rui et al. [84] fabricated SS hollow fibres in a variety of sintering atmospheres (air, carbon dioxide, nitrogen, helium, hydrogen). The prepared SS hollow fibres (Figure 1 (1)) showed different exterior colours, which was attributed to the formation of different composition of membrane materials under various sintering atmospheres, and the hollow fibres prepared under hydrogen displayed the largest shrinkage among the fabricated hollow fibres (Figure 1 (2)). They found that sintering with hydrogen can assist in removing the polymer binder and could eliminate oxidation on the metal surface. The sample (mean pore size 2.1 µm) sintered at 1100 °C in H_2_ showed a bending strength of 384 MPa, which was greater than that of most of ceramic hollow fibres reported in the literature [85,86,87,88]. Wang et al. [83] developed SS hollow fibre membranes with a three-channel structure (Figure 2) and demonstrated a further improvement in the mechanical properties of the membrane. Most recently, Li’s research group used this technique to develop dual-layer composite hollow fibres [89]. The fabricated dual-layer SS/SS-ceramic composite hollow fibres have an inner SS layer and an outer layer composed of a mixture of SS and yttria-stablilised ZrO_2_ (YSZ) (Figure 3), with a mean pore size of ~0.3 µm and an enhanced bending strength (>400 MPa). Although the mechanical strength has been much enhanced, the SS hollow fibres still display relatively large pores (in the µm range) [78,83,84], which has restrained their practical applications such as MF. In addition, very limited studies [89] have been carried out to develop SS hollow fibres with smaller pore sizes. The influence of sintering conditions including temperature, time, and atmosphere on SS hollow fibres is still not well known [73]. Further work is necessary to precisely control sintering conditions by carefully considering the properties of the materials used (e.g., size and size distribution of particles, binder thermal stability), coupled with the atmosphere, temperature, and time used for the sintering process [73]. Using precursor materials with smaller particle size (e.g., nano-particles) is also necessary to possibly achieve smaller pore (e.g., sub-micron) metal membranes for MF applications [78].

Supported metal membranes are commonly fabricated by coating a thin layer of ultrafine metal oxides such as TiO_2_, Al_2_O_3_, or ZrO_2_ onto a porous metal substrate (generally porous stainless steel) followed by a sintering process. The Sol–gel process is one of the conventional methods used to coat the thin layer onto the porous SS substrate [90]. Li et al. [90] used this technique to introduce an α-Al_2_O_3_ intermediate layer onto a porous SS substrate, and then they fabricated a top layer of TiO_2_, SiO_2_, or TiO_2_-SiO_2_ on the α-Al_2_O_3_ intermediate layer by the same technique. By using this 2-step approach, they successfully reduced the pore size from 1.5 µm (porous SS substrate) to 0.7 µm (α-Al_2_O_3_ intermediate layer), and further down to ~0.3 µm (top layer of TiO_2_, SiO_2_, or TiO_2_-SiO_2_).

**Figure 4:**
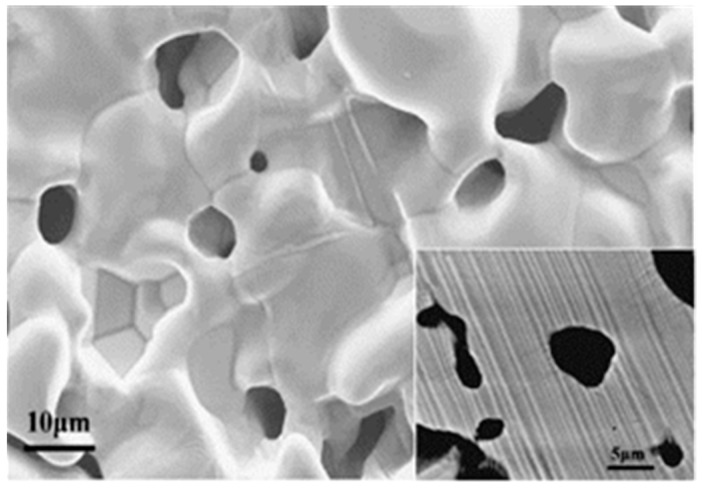
SEM image of the Ti–48Al–6Nb alloy coating surface (inset presents the cross-section of the coating skeletons). Reprinted from “Innovative fabrication of Ti–48Al–6Nb porous coating by cold gas spraying and reactive sintering” [94], © 2012, with permission from Elsevier.

**Figure 5:**
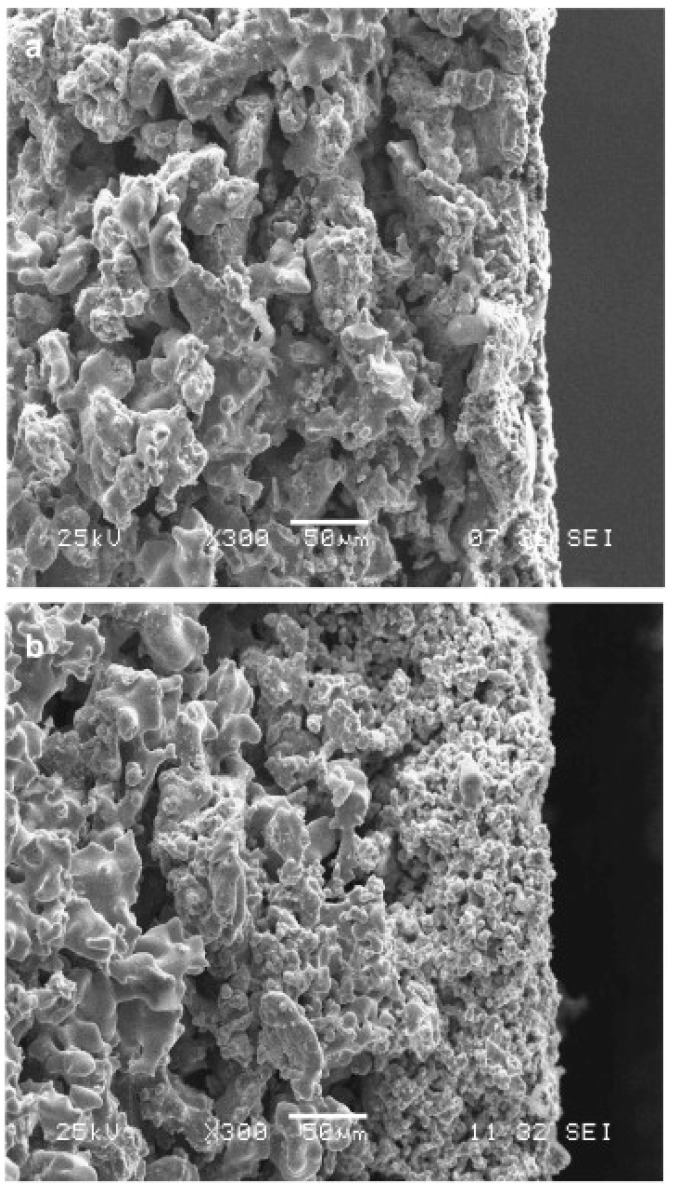
SEM images of cross-section of Fe–Al-alloy supported membranes with different thicknesses: (**a**) 10 μm and (**b**) 120 μm. Reprinted from “Development of a new graded-porosity FeAl alloy by elemental reactive synthesis” [93], © 2009, with permission from Elsevier.

Metal membranes supported on porous metal alloys substrates such as Ti–Al or Fe–Al have also been studied [91,92,93,94] as potential functional materials for molecular separation at high temperature or in corrosive environments. Wang [91] fabricated a porous Ti–Al-supported Ni using a dip-coating process followed by sintering. The prepared Ni/Ti–Al membrane showed an average pore size of 0.83 µm and a pure water flux of 6782 L·m^−2^·h^−1^. Zhou et al. [92] prepared a MF TiO_2_ membrane supported on a planar porous Ti–Al alloy by combined electrophoretic deposition and dip-coating process followed by sintering in argon atmosphere at 1050 °C. The fabricated TiO_2_/Ti–Al showed a defect-free surface and an average pore size of 0.28 µm, and achieved a pure water flux of 3037 L·m^−2^·h^−1^. Yang and co-workers [94] developed a Ti–48Al–6Nb porous MF coating (Figure 4) on a high Nb–TiAl porous alloy support using cold gas spraying followed by reactive sintering. The porous high Nb–TiAl alloy support used in their study had the same composition as the coating and was prepared using the same procedures as described by Wang et al. [95]. The prepared porous Ti–48Al–6Nb coating had an average pore size of 1.8 μm, and showed high permeability and sufficient strength for potential MF applications in extreme environments. Shen et al. [93] fabricated Fe–Al alloy supported membranes with graded pores (Figure 5) by Fe and Al elemental reactive synthesis. An Fe–Al alloy with large connecting open pores and permeability used as a support was formed using powder metallurgy techniques. The coating was achieved by spraying slurries containing mixtures of Fe particles and Al particles (both 3–5 μm in diameter) onto Fe-Al alloy support followed by sintering at 1100 °C. The prepared membranes showed a mean pore size of coating is 2.5 μm (maximum pore size 6 μm, minimum pore 1.7 μm).

## 3. Commercial Porous Metal Membranes

There are more than 30 companies producing metal membranes [4]. Top sellers of global metal membranes market include GKN Sinter Metals, Metalmembranes, Sterlitech Corporation, Pall Corporation, Porvair Filtration Group, and Mott Corporation. Most of the top players are located in United States, Europe, and Japan. Materials used for producing commercial metal membranes include SS, Ti, Ni, metal alloys (e.g., Hastelloy, Alloy 20), and SS-supported metal oxide (e.g., TiO_2_, ZrO_2_). Commercial porous metal membranes generally come in three basic configurations—tubular, disc, and flat sheet. Table 2 lists a wide range of porous metal filters/membranes that are currently commercially available. It can be seen from Table 2 that sintering is the main technique used for fabrication of commercial porous metal filters/membranes.

### 3.1. Porous Metal Membranes Based on Stainless Steel (SS)

**Figure 6:**
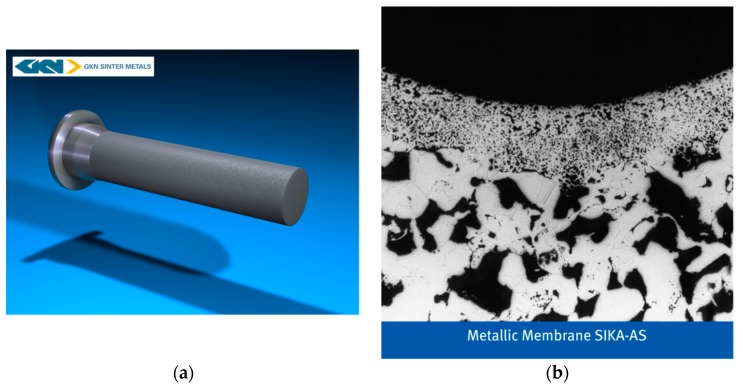
SS-based porous metal membranes from GKN [96]: (**a**) high porous SS filter (the outer surface of the filter has an effective layer of 0.2 mm with a typical length of 1500 mm and an outer diameter of 64 mm, flow configuration: outside-inside); (**b**) metal membrane coating (~200 µm thick) on porous SS filter. Reproduced with permission from GKN.

Although a wide range of materials is available for production of commercial porous metal membranes, the majority of current commercially available porous metal membranes are made of SS or based on SS. Most of the top players in the global metal membranes market, such as GKN Sinter Metals, Pall Corporation, Mott Corporation, and Porvair Filtration Group, provide SS or SS-supported porous metal membranes. Figure 6 shows a typical porous SS filter and SS-supported metal membrane from GKN [96]. The highly porous SS filter (Figure 6a) is prepared by cold isostatic pressing followed by sintering, while the metal membrane (Figure 6b) is fabricated by coating a layer of extremely fine metal powder onto a support of coarser porosity (e.g., sintered SS filter (Figure 6a)) followed by sintering process. SS or metal alloys such as Inconel 600, Inconel 625, and Monel 400 can be used as a membrane coating material. Pall Corporation manufactures tubular SS filters such as PMM^®^ [32] and AccuSep™ [31] by sintering, and SS-supported ZrO_2_ [101] by depositing a yttria stabilised zirconia onto a Pall AccuSep™ SS tubular element followed by sintering. Mean pore size of 0.1 µm can be achieved by coating a ZrO_2_ membrane layer onto an AccuSep™ SS tubular support. Mott Corporation provides standard porous SS filter elements [76] for a variety range of applications such as chemical processing, food and beverage and wastewater treatment, and also developed sterilising grade all-metal (e.g., SS) filtration membranes [107] for medical filtration applications. The all-metal sterilising grade membrane meets or goes beyond a 200 nm filtration challenge and meets ASTM F838-05 for bacterial retention. Porvair Filtration Group also fabricates porous metal materials and structures (Sinterflo^®^) from finely divided metal powders such as SS for filtration applications in a wide range of industries [29]. Both Graver Technologies, LLC (Scepter^®^) [102] and Hyflux (FerroCep^®^) [103] offer SS-supported TiO_2_ membranes (nominal pore size ~0.1 µm or ~0.02 µm) for many industrial filtration (MF, UF) needs. Both Scepter^®^ [102] and FerroCep^®^ membranes [103] are fabricated using a similar approach, in which a TiO_2_ membrane layer is permanently bonded to the porous SS substrate by sintering. The sintering technique can create a smooth and foulant-resistant membrane layer with sharp pore size distribution suitable for precise MF and UF separations.

### 3.2. Porous Metal Membranes Based on Alloys

Apart from SS, a variety range of metal alloys have also been used for the fabrication of commercial porous metal filters/membranes by many companies due to their unique characteristics including low density, good thermal conductivity, high mechanical strength, and corrosion resistance. The majority of metal alloy based porous metal filters/membranes on the market are made from nickel-based alloys including Hastelloy, Inconel, and Monel 400 (Table 2). GKN Sinter Metals, Pall Corporation, Mott Corporation, and Porvair Filtration Group are the main user of nickel-based alloys for their membranes. Other metal alloys such as bronze and FeCrAl alloy are also used for manufacturing porous metal filters/membranes (Table 2). GKN uses nickel-based alloys (e.g., Hastelloy C276, Hastelloy X, Inconel 600, Monel 400) as standard materials for its SIKA-R…IS sintered porous metal filters [96] and the SIKA-R…AX series [97]. Inconel 600 and Monel 400 are also used by GKN for manufacturing SIKA-R…AS high porous asymmetric metallic membranes [96], while bronze (89/11 AK) powder is used for its SIKA-B high porosity sintered elements [99]. Mott [76] uses a variety of nickel-based alloys including Hastelloy B, Hastelloy B-2, Hastelloy C22, Hastelloy C276, Hastelloy N, Hastelloy X, Inconel 600, Inconel 625, Inconel 690, Monel 400, Nickel 200, and Alloy 20 for the fabrication of sintered porous metal filter elements. Porvair Filtration Group offers a broad range of materials (Inconel 601, Inconel 600, Hastelloy X, Monel, NiCrMo Alloy 59, FeCrAl Alloy, Bronze) for its Sinterflo^®^ range [29]. Pall also uses Hastelloy X and Inconel 600 for manufacturing AccuSep™ [101] membranes.

### 3.3. Porous Metal Membranes Based on Other Metals

**Figure 7:**
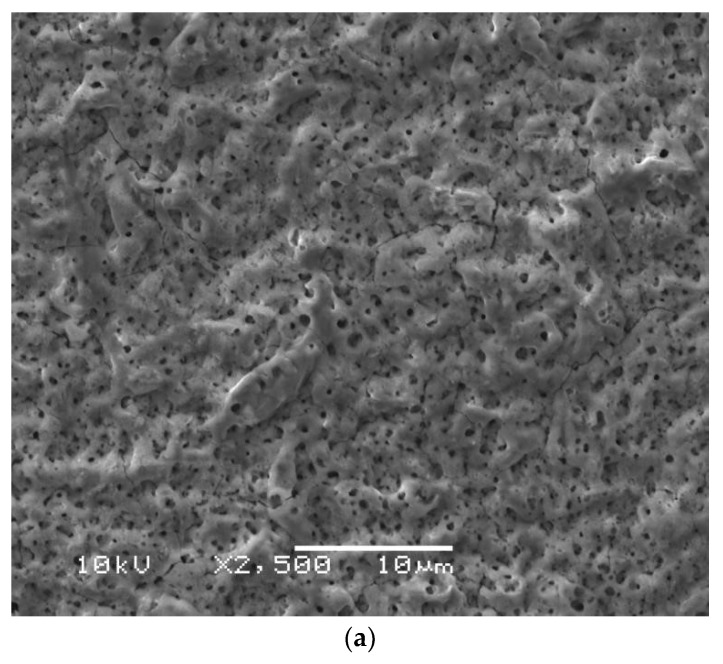

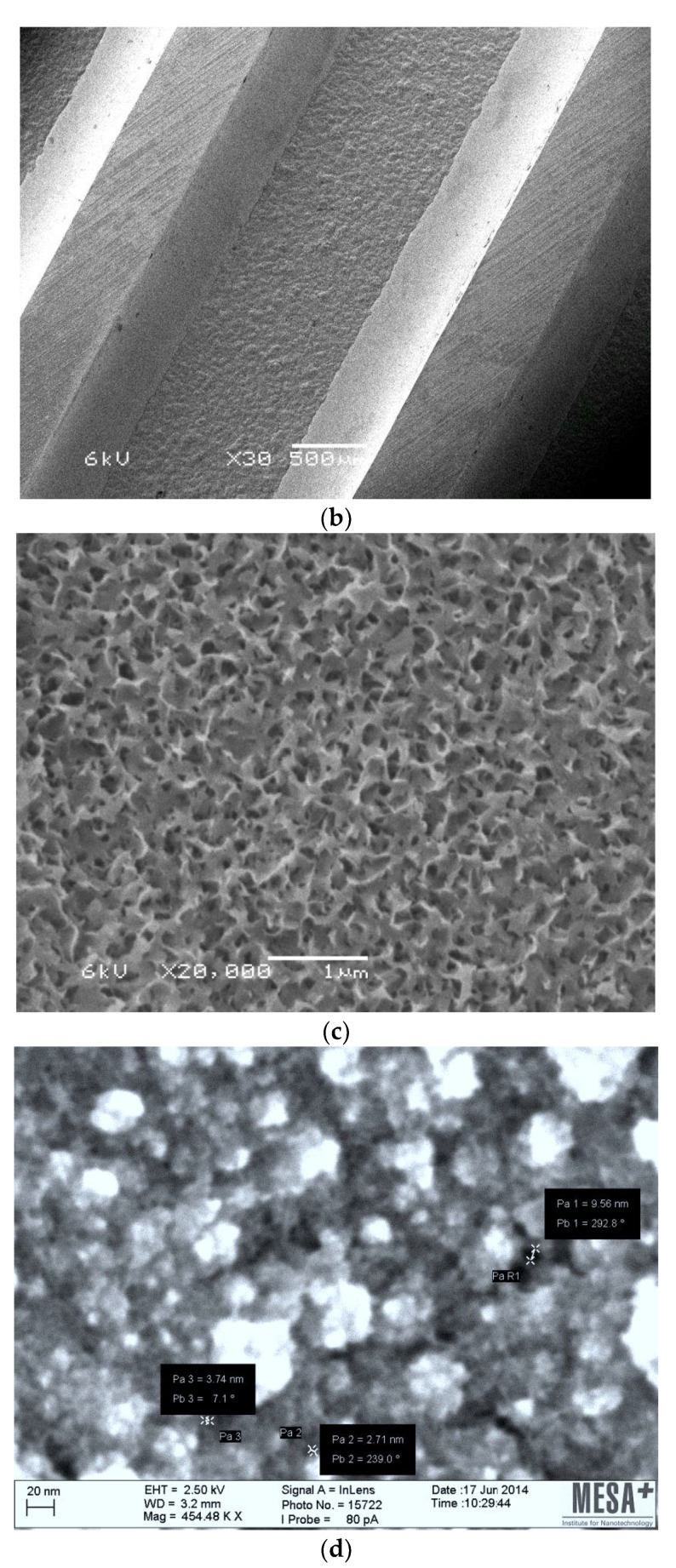
SEM images of membranes developed by Metalmembranes: (**a**) The outer surface of the ceramic layer before electrochemical machining (ECM), (**b**) after ECM flow channels in aluminium membrane, showing the metal support bars and the inner surface of the ceramic layer, (**c**) magnification of the pores (100–150 nm) on the inner ceramic layer of an aluminium membrane, (**d**) magnification of the pores (2–10 nm) on the inner ceramic layer of a titanium membrane. Reproduced from Novel hybrid ceramic metal membrane [108] with permission from Metalmembranes.

Other metals used for manufacturing commercial porous metal filters/membranes include titanium, nickel, silver, and aluminium. Titanium has superior industrial performance properties such as exceptional strength, fouling-resistance, non-corrosive, pH-resistant, and it is able withstand temperature and pressure fluctuations. This makes it ideal for use as a separation material. GKN uses Ti as a standard material for production of SIKA-R…IS [96] and SIKA-R…AX [97] sintered high porosity metal filter elements. Mott also uses Ti for sterilising grade all-metal filtration membranes [107] in addition to SS. AMS [106] also offers titanium filtration membranes for wastewater treatment. Metalmembranes also uses titanium as a base material to produce supported metal oxide membranes [104,108]. Metalmembranesdeveloped a unique process to produce metal membranes from tough metal materials, e.g., titanium or aluminium. The membranes have a highly porous metal oxide (ceramic) layer coated on one surface of the support material (titanium or aluminium) by a plasma oxidation process, while the other surface of the support consists of porous metal (titanium or aluminium) with straight, channel structures created after the metal was partially removed by an electrochemical machining (ECM) process (Figure 7) [108]. The membrane pore size is tunable from 150 nm (aluminium) to 10 nm (titanium), making the membranes especially suitable for detection and diagnostic applications [104,108]. Other metals such as Ni are used by Pall [101] to produce membranes for gas separation, and silver is used by Sterlitech for its Sterlitech™ membrane filters [105].

## 4. Applications of Porous Metal Membranes

Metal membranes have been widely used for food and beverage applications [8,27,30,76,106,109,110]. However, the first large-scale use of microporous metal membranes was back in the 1940s for gas separation of U235F from U238F as part of the Manhattan project [111]. Porous metal membranes were later developed for use in food, drug, and beverage industries in the 1960s [8]. During the past few decades, various new porous metal membranes have been developed for use in not only food/beverage applications, but also in a range of other fields such as chemical/petrochemical processing, liquid/solids, and gas/solid separation, and biofuel processing [23,27,29,30,31,76,106,110]. Porous metal membranes have also been used for medical filtration applications [107], solid-phase extraction [112], catalyst recovery [113], and a number of studies related to water applications [10,11,12,13,14,15,16,17,18,19,20,106,114]. However, commercially available porous metal membranes are mainly used to filter solid particles from liquids, and the application of porous metal membranes in water treatment is reviewed here.

### 4.1. Porous Metal Membrane Filtration

Metal membranes have been studied for their filtration in a number of water applications such as drinking water treatment [10,21], rainwater purification [114,115], wastewater reclamation [11], and the treatment of wastewater from industries (e.g., meat processing) [116]. Leiknes and co-workers [10,21] studied the feasibility and potential of using microfiltration metal membranes (pore size 0.2 µm, Hitachi Metal Ltd., Tokyo, Japan) in a submerged membrane configuration with coagulation pre-treatment for producing drinking water. They achieved >95% true colour removal, ~85% removal of UV-absorbing compounds, 65–75% TOC reduction, and <0.2 NTU turbidity. These results showed that the process of using MF metal membranes with coagulation pre-treatment offers good technical potential as an alternative to the commonly used methods (e.g., sand filtration in coagulation/direct filtration using polymer membranes) for drinking water production. However, process costing and comparison with other membrane processes is needed for further feasibility studies. Kim, et al. [114,115] used metal membranes (pore size of 1 µm and 5 µm, FiberTech, Seoul, Korea) in combination with ozonation to remove contaminants from rainwater. The metal membranes demonstrated excellent chemical stability when used with ozone and high efficiency for the reduction of microbial and particulate pollutants in rainwater. Metal membrane MF was also integrated with electrodialysis for wastewater reclamation [11]. This integrated system used a metal membrane (made from SS, effective membrane area 0.12 m^2^, pore size 0.1 µm) supplied by Hitachi Metals Co., Ltd., Tokyo, Japan. The integrated system achieved good rejections for bacteria, suspended solids and ionic nutrients, and stable water quality over 6 months of operation. Cross-flow filtration systems incorporating AMS tubular titanium membranes with 0.2 µm pores have also been attempted on a range of feed solutions [106]. The system is able to provide liquid/solid separation to 0.05 µm (UF) under a variety of testing conditions (e.g., temperature, pressure, pH). The performance test of the AMS 0.2 µm titanium membrane filtration system on feed solutions from the textile, poultry, dairy processing, and meat processing industries has demonstrated that a single pass through the 0.2 µm titanium membrane system achieved a reduction of 96.5–99.9% for suspended solids, fats, oil and grease, and 36.3–96.5% for COD [106]. Titanium membranes can be cleaned easily by caustics, HNO_3_, NaClO or steam, and membrane flux can be recovered upon cleaning.

Most recently, metal membrane filtration (MMF) was used to remove fats and COD from wastewater from meat rendering operations as pre-treatment for membrane distillation (MD) [116]. Meat processing produces stick water containing high amounts of fats and proteins that foul and wet hydrophobic polymeric MD membranes. The AMS BT500-65 MMF system, consisting of a pack of metal membranes (pore size 0.5 µm, active area 0.26 m^2^), was supplied by AMS, Australia. The filtration experiments were conducted in a cross-flow setup with the feed solution being fed on the inside and permeating to the outside of the membrane. MMF pre-treatment removed 92% of fats and 35% of proteins, and led to superior MD performance, demonstrating the potential of metal membrane filtration for fat removal and recovery. Flux reduction during MD of the MMF pre-treated stick water was minimal compared to the MD of raw stick water, indicating that MMF pre-treatment can significantly reduce fouling of the polymeric MD membrane. The flux of the metal membrane can be recovered upon cleaning using caustics.

### 4.2. Porous Metal Membrane Contactors for Membrane Evaporation

**Figure 8:**
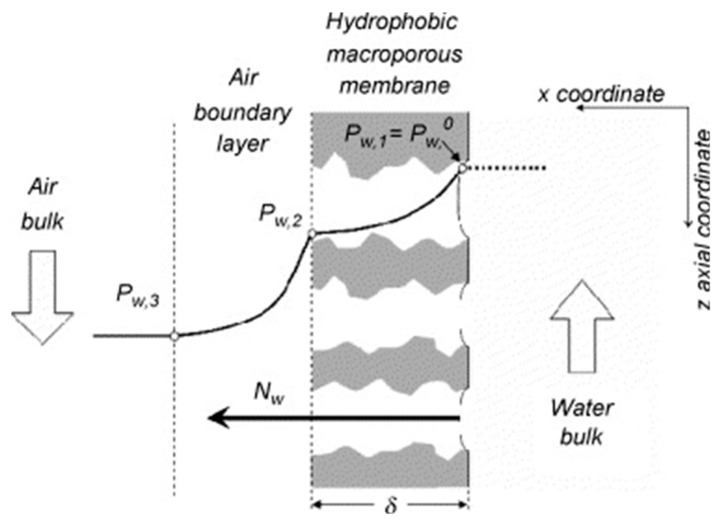
Water vapour pressure profiles in the membrane evaporation (ME) process. Reprinted from “Study of a new membrane evaporator with a hydrophobic metallic membrane” [19], © 2006, with permission from Elsevier.

Membrane contactor processes use a porous membrane to carry out aqueous solutions concentration. There are two main membrane contactor processes: osmotic evaporation and membrane distillation. Osmotic evaporation [117] is a process in which a porous membrane is used to separate feed and osmotic solution. The driving force of this technique is the difference of water vapour pressure between both sides of the membrane. MD [118] is another concentration process based on the use of a porous membrane as a barrier between the feed and the distillate. The driving force of this technique is the vapour pressure difference caused by the temperature difference between both sides of the porous membrane. These two processes possess a higher selectivity for non-volatile compounds (100% retention of ions, macromolecule, colloids, and cells) than other membrane processes, and less energy consumption than the traditional water distillation in a single step [19]. However, osmotic evaporation with metal membranes has corrosion problems due to the use of brine, and MD requires relatively high temperatures. These drawbacks have limited the generalisation of these two techniques for the concentration of fluids [19]. Membrane evaporation (ME) represent an emerging technology in which an aqueous solution is concentrated by continuous evaporation through a porous and heat-conductive membrane [19,119]. This process is midway between osmotic evaporation and MD and governed by the difference of water vapour pressure between the feed and a downstream flow of dry gas (generally air) (Figure 8) [19]. In contrast to MD, the flow of water vapour in ME process is not condensed, but taken away by the extracting phase. ME can be operated at room temperature, which possesses the potential of ME for heat-sensitive solutions [19].

Porous metal membranes have recently been studied for use as membrane contactors for the ME process [19,20,119,120,121]. Sanchez’s group has developed a complete mass and heat transport model [121] and tried a number of metal membranes for the ME process [19,119,120]. Their initial work [19,119] was conducted on SS sheet membranes (M020 and FH050, Pall) modified by a silicon-type material to obtain hydrophobic properties. The results obtained from this initial work validated the concept of ME process on a metal membrane contactor. The most attractive evaporation fluxes were achieved on FH050 which has larger pores (5 µm) and higher porosity (75%) compared to M020 (pore size 2.6 µm and porosity 30%). After a complete mass and heat transport model [121] was developed, they conducted further studies on the influences of a range of operating conditions such as air velocity, feed temperature, and air pressure on the ME process [120]. Three different flat macroporous metal (ASI 316L stainless steel) membranes were evaluated in their study, and the results revealed that higher air velocity and higher temperature of the feed solution resulted in greater water evaporation flux. The study also found that the geometry of the experimental flat sheet device restricted the range of operating conditions, and suggested changing to a configuration with multi-tubular membranes. Using porous, hydrophobic metallic membranes allowed the heat to be applied directly to the membrane, and then it was transferred to the liquid gas interface [19], thus leading to significant energy savings [19,20]. However, future work is necessary to compare the metal membrane-based ME processes with those using polymeric membranes. Analysis on the thermal efficiency of these systems is also needed for comparison with MD processes.

### 4.3. Porous Metal Membrane Bioreactors

The membrane bioreactor (MBR) has been widely used in wastewater treatment. By replacing the secondary sedimentation tank used in the conventional activated sludge process (CASP) with a membrane separation unit, MBR has overcome many disadvantages of CASP, including poor solid-liquid separating efficiency, low levels of mixed liquor suspended solids, and a slow biochemical reaction rate, and this results in good quality effluent [122]. Since a membrane is the key component in the MBR, the material used for membrane fabrication is critical. Most membranes used in MBR are made of low-cost organic materials; however, it is well known that they are weak and cannot withstand prolonged exposure to extreme acidity or alkalinity, temperature, and high-pressure operations. Inorganic membranes made from ceramic or metals have also been studied for the MBR. Zhang, et al. [13] studied the use of a flat SS membrane (nominal pore size 0.2 µm, Hitachi Metals, Tokyo, Japan) in an aerated submerged MBR for the treatment of synthetic domestic sewage. The metal membrane achieved a steady flux of >17 L·m^−2^·h^−1^ and a mean COD removal efficiency of 97% (typically 96–99% from polymer membrane-operated MBRs [123]).

There are more cleaning methods available for metal membranes than polymer membranes [13]. Zhang et al.’s study demonstrated that membrane fouling could be effectively eased using on-line backwash and intermittent running mode, and could almost entirely be eliminated using off-line cleaning by 0.25 wt % NaClO solution. Xie, et al. [15] also used a stainless steel sheet membrane (pore size 0.4 µm) supplied by Hitachi Metals, Tokyo, Japan in a submerged MBR for treatment of synthetic domestic sewage under two different modes: an aerobic membrane bioreactor (AMBR) and an anoxic/aerobic membrane bioreactor (A/O-MBR). The experiment was continuously run for 270 days, and the AMBR achieved a mean removal of 96.7% for COD and 32.1% for TN, while the A/O-MBR showed a mean rejection of 92.2% and 72.4% for COD and TN respectively. The Hitachi Metals flat stainless steel membrane (pore size 0.2 µm) was also studied for a calefactive aerobic MBR for treatment of simulated distillery wastewater with a COD of ~1000 mg·L^−1^ at 30–45 °C [14]. The process achieved a mean removal efficiency of 94.7% for COD, and 84.4% for TN. These results implied that metal membranes could be an alternative to organic membranes for MBRs for wastewater treatment. Metal membranes offer some advantages over polymer membranes, such as high mechanical resistance, extended working lifetime, and easy cleaning, especially their high heat durability, making them more attractive for wastewater treatment at higher temperature [14]. However, limited work so far has been carried out on the use of metal membranes in MBR processes due to their relatively high cost [13].

### 4.4. Photocatalytic Metal Membranes

**Figure 9:**
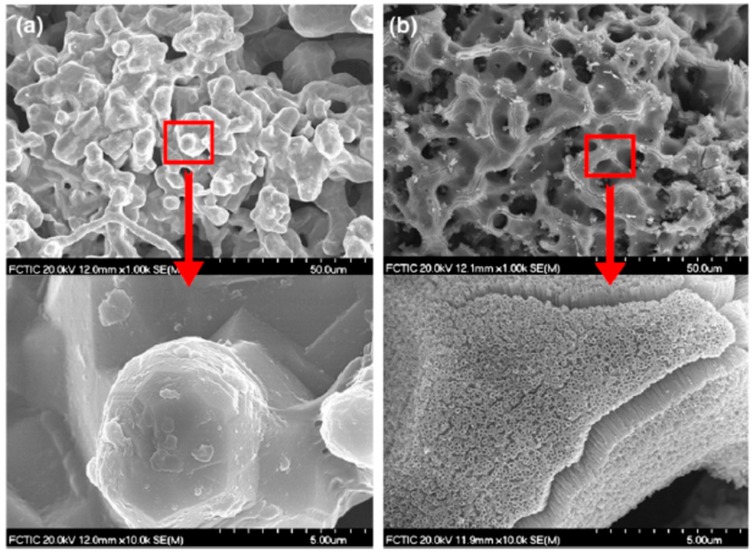
FE-SEM measurements on (**a**) an original Ti membrane filter and (**b**) an anodised TiO_2_ membrane (the diameter of nanotubes exceeded the range of 25–75 nm and a wall thickness of ~15–20 nm). Reprinted from “Fabrication and photocatalytic activity of a novel nanostructured TiO_2_ metal membrane” [130], © 2011, with permission from Elsevier.

Photocatalytic technologies have been extensively studied for the removal of organic contaminants in wastewaters [124,125,126,127]. However, the use of aqueous suspensions of photocatalysts (e.g., TiO_2_) requires separation following treatment, which has constrained their practical applications in water treatment. Many studies have been carried out to develop TiO_2_ thin films and membranes for use in a range of applications [128,129]. Recently, Choi, et al. [130] used an anodisation method for the development of self-organised, nanostructured photocatalytic TiO_2_ metal membranes with the combined advantages of TiO_2_ photocatalysis and membrane filtration. In their study, nanostructured photocatalytic TiO_2_ metal membranes (Figure 9) were fabricated on microporous (nominal pore size 0.5 µm) tubular-type pure titanium filters, and their photocatalytic activity was evaluated for decomposing organic contaminants (humic acid). The prepared TiO_2_ metal membranes demonstrated high efficiency in the photocatalytic decomposition of organics, and good membrane permeability. Photocatalytic TiO_2_ metal membranes are able to overcome the photocatalytic activity and long-term durability limitations of the catalyst-polymer composite membranes that tend to result in the degradation of the polymer matrices due to the formation of hydroxyl radicals produced from the photocatalyst (e.g., TiO_2_). However, future work needs to be focused on reducing the cost of photocatalytic TiO_2_ metal membranes to make them more compatible with the membranes made from other materials (e.g., composite, ceramic).

### 4.5. Catalytic Metal Membranes

Catalytic membranes containing the catalytic metals (e.g., Pd, Cu) have been studied for the denitrification (hydrogenation of nitrate) [17,131,132] and oxidation of organic components [133] in water. Catalytic hydrogenation reduces nitrates into harmless nitrogen; thus they can be used as an alternative and eco-friendly approach for water denitrification. In 1989, catalytic hydrogenation using bimetallic Pd-based catalysts was proposed as a new process to remove nitrates in drinking water [134]. Supported-Pd catalysts with Cu or Sn acting as promoter have been identified to be the most suitable systems for the reduction of nitrate in aqueous solutions [131,132,135,136]. Nitrate conversion can be dramatically enhanced by using supported Pd catalysts with promoters. For example, Ilinich and co-workers [17] compared the catalytic behaviour between Pd–Cu catalysts supported on γ-Al_2_O_3_ granules and the catalytic membranes (macroporous ceramic (Al_2_O_3_) membrane containing Pd–Cu), and found that forced flow of nitrate-containing water through the catalytic membrane promoted a multi-fold increase in the catalytic activity. Significant improvement in catalytic activity by using the catalytic membranes (e.g., ceramic membranes deposited with Pd and Cu or Sn) was also confirmed by other research groups [131,132,135]. Despite porous catalytic membranes significantly improving catalytic activity of the denitrification process, a problem with the formation of ammonium due to over-hydrogenation still exists. Catalytic nitrate reduction process still cannot achieve very high selectivity for nitrogen without substantially losing activity [131]. The catalytic membrane process has also been recently tested for the oxidation of aromatic components in water-based and seawater-based synthetic solutions by Kumakiri and co-workers [133]. Pt as the catalyst was deposited onto a porous ceramic tube supports with a top zirconia layer (thickness 6 µm, pore size 50 nm). The catalytic membrane process achieved a 97% removal for C2-phenol (catalytic oxidation removed 72%, while evaporation contributed to 25% of the removal). Although these results showed that the processes using catalytic metal membranes seemed promising for both denitrification and catalytic oxidation of aromatic components in water, operating costs were high as the catalysts are made from noble metals (e.g., Pt, Pd), and they need to be replaced once they have lost their catalytic activity. Therefore, from an economical point of view, these catalytic metal membranes need to be considered for higher value applications than the above mentioned purposes. Most recently, Allioux and co-workers [137] developed nickel (Ni) and nickel-copper (NiCu) alloy hollow fibre membranes for the electrocatalytic degradation of small organic molecule contaminants from model wastewater effluents (salicylic acid (SA)). The novel metal hollow fibre membranes were found to be highly stable and reusable, while the kinetics of SA electro-oxidation were 9–20 times greater than with pure platinum wire electrodes.

## 5. Research Opportunities

An increasing number of studies related to porous metal membranes has underlined the potential of using these membranes more widely in separation applications. Porous metal membranes are more suitable and competitive than the existing membrane materials for the purification of abrasive or corrosive streams where polymers degrade too rapidly or ceramics are too brittle. However, current commercial porous metal membranes are mainly produced by sintering process, which has restricted their practical applications, such as in water applications, as the sintering technique typically produces large pore size (>1 μm), limited pore connectivity and relatively low porosity (<50%) [28]. Smaller pore size metal membranes, therefore, offer clear opportunities that should be thoroughly investigated to reveal their full potential. Size exclusion is directly related to the membrane pore size, so nanoscale pores should be preferred to achieve selective rejection of smaller particles that cannot be otherwise removed from the contaminated stream. In addition, backwashing of a filter cake from a stiff porous material (e.g., metal) is known for being enhanced compared to that of a soft material (e.g., polymer). The smaller pores of metal nano-filters will provide enhanced pore cleaning, as opposed to pore constriction and the difficult cleaning issues reported for polymeric membranes.

Recent work has demonstrated the feasibility of approaches to design metal nanoscale porous networks including sintering of nano-composites [60], metal-terminated block-copolymer self-assembly [33,71], metal nano-foaming, de-alloying [138], electroless deposition [139,140], electro-spinning of metal nano-fibre webs [40,42], or metal oxide reduction into porous nano-structures [141]. Electro-spinning is of particular interest as it provides very high throughput and permits for the fabrication of a large range of fibre orientations and diameters, leading to a range of pore size and porosity [142]. Electroless deposition is also a very promising technique for producing functional nano-porous networks as deposition of metal can be achieved onto a large variety of supports to offer specific pore morphology [62]. The recent investigation of electroless deposition of stable metal alloys may also lead to multi-scale porous structures beneficial to the treatment of complex industrial waste solutions [143]. Most of the techniques mentioned above are versatile and scalable, and therefore offer clear opportunities to scale up for the fabrication of membranes. However, their commercial production has yet to be proven.

The accurate control of the surface energy is also a recurrent key point in membrane science to optimise both selectivity and permeation. The functionalisation of metal surfaces has been a strong research area over the past 20 years. Routes to adapt surface energy and functional groups to significantly reduce corrosion and alter hydrophobicity of metal surfaces have been successfully demonstrated [9,144,145]. However, research in the surface chemistry of porous metal membranes is relatively poorly documented. The optimisation of the surface properties of commercially available metal membranes has yet to be carefully examined. A fundamental understanding of the chemistry between the membrane surface and the contaminants in solution also needs to be improved. Therefore, there are opportunities for the development of new processes for preparation of highly porous metal membranes with tuned nano-scale pore morphology and chemistry.

Although the commercially available porous metal membranes are claimed to have high corrosion resistance and can handle wide-ranging corrosive environments, little work has been carried out on corrosion resistance studies on porous metal membranes [146]. It is known that a porous material can be more easily corroded than a corresponding solid material of the same chemistry due to its high exposed surface area [146]. More work needs to be done to enhance the fundamental understanding of corrosion mechanisms on metal membranes, and develop new materials with enhanced corrosion resistance property.

Cost is another important area that needs to be explored. As mentioned earlier, high capital costs are one of the main drawbacks that hinders the wide use of inorganic membranes [3]. Therefore, in order to target niche markets in water applications, further research in to the feasibility of novel fabrication routes is needed for economic production of nanoporous metal membranes to reduce capital and maintenance costs.

## 6. Conclusions and Prospects

In this review, we have presented an overview of the state of the art in preparation and potential applications of porous metal membranes. Although most commercially available porous metal membranes are made by sintering or foaming methods and have been mainly used for the filtration of liquids to remove the solid particles, porous metal membranes offer some advantages (e.g., high mechanical strength, high temperature and pressure capability) over polymeric membranes and have a great potential in water applications. Despite porous metal membranes having been less well studied compared to polymeric and ceramic membranes, metal membrane surfaces offer a versatile array of potential chemistries, and they possess great prospects through novel fabrication and functionalisation routes such as de-alloying and electroless deposition. Recent research on the functionalisation of metal nano-particles has also opened routes for the precise modification of metal surfaces, leading to the control of membrane surface energy and hydrophilicity. For porous metal membranes to be widely used across a number of applications, in particular for niche markets in water applications, the preparation of functional nanoscale porous metal membranes is desired, and capital and maintenance costs need to be significantly reduced by using economical approaches for fabrication. Significant breakthrough can occur if sufficient efforts are focused on the combination of these different areas of investigation.

## Figures and Tables

**Table 1:** Techniques used for the fabrication of porous metal frameworks with different pore size range.

Pore Size	Techniques	Advantages	Disadvantages
>100 µm	Casting [9,22]	Close control of the pore size distribution.	Inadequate interconnectivity of the pores.
	Electroplating [22,23]	High efficiency for the fast processing of rough metal coatings.	Required to use the surface as an electrode, which leads to pore filling and clogging within porous structures, thus substantially reducing the surface porosity and pore density.
	Chemical vapour deposition (CVD) [9,24,25,26]	Can produce a thin imprint that accurately follows the topography and morphology of the substrate.	Usually only used to coat thin layers of pure metals onto the substrate.
1–100 µm	Thermal sintering [27,28,29,30,31,32]	Mature technology, easily scale up, cheap to process.	Low pore connectivity and limited porosity.
50 nm–1 µm	Template-directed synthesis [33,34]	Can deposit metals onto a template structure of the desired pore size (e.g., colloidal arrays).	The uniform deposition of metals into colloidal arrays is challenging.
	De-alloying [8,35,36,37,38,39]	Could lead to very homogeneous structures with narrow pore size distribution.	Difficult to form ultra-thin films of fine grain size alloys.
	Electro-spinning [40,41,42,43,44,45,46]	High up-scalability and low cost.	Mechanical strength needs to be enhanced by post-treatments.
	Wet casting/coating [47,48,49]	Easy to implement.	Relatively large pores (~1 µm) may exist in the final membranes.
	Ink-jet printing [50,51,52,53,54,55,56,57,58,59]	Cost effective, form multi-material components, precisely fabricate intricate layers, able to cover 3D surfaces.	Need post-treatments, still immature.
	Electrical sintering [60,61]	Creates finer structures than thermal sintering.	Only can achieve very thin films (<250 nm).
1 nm–50 nm	Electroless deposition [62,63,64,65,66,67]	Highly controllable structures with nano-scale pore distribution, able to simultaneously co-deposit multiple metals.	Very low deposition rate, a careful analytical control of the plating bath is required, high cost.
	Block co-polymer (BCP) [68,69,70,71]	Fine control of the nanoparticle distribution, can result in highly crystallised and ordered structures.	Using expensive BCPs prohibits their expansion to a large scale.

**Table 2:** Summary of commercial porous metal filters/membranes.

Manufacturer	Trademark/Brand	Material	Technique	Configuration	Pore Size (µm)	Main Applications
GKN	SIKA-R…IS [96]	SS, nickel-based alloys, Ti	Cold isostatic pressing–sintering	Tubular	0.5–200	Catalyst separation and recovery Refinery applications Gas filtration (e.g., hot off-gas or superheated steam) Aerosol separation
GKN	SIKA-R…AS [96]	SS, nickel-based alloys	Coating–sintering	Tubular and disc	0.1–3	Catalyst separation and recovery Refinery applications Gas filtration (e.g., hot off-gas or superheated steam) Aerosol separation
GKN	SIKA-R…AX [97]	SS, nickel-based alloys, Ti	Co-axial pressing–sintering	Disc, cylinder, plate, cone	0.1–200	Polymer filtration Gas filtration (e.g., hot off-gas or superheated steam) Liquid filtration (e.g., catalyst recovery) Sparging
GKN	SIKA-FIL [98]	SS, FeCrAl alloy	Powder metallurgical process–soft sintering	Sheet	1–100	Aerosol separation Polymer filtration Gas and liquid filtration Hot gas filtration
GKN	SIKA-B [99]	Bronze	Moulding–sintering	Disc, cylinder, plate, cone	8–200	Aerosol separation Polymer filtration Gas filtration (e.g., hot off-gas or superheated steam) Liquid filtration (e.g., catalyst recovery) Autogenous welding (as flame arrestors)/Explosion protection Sparging
Pall	PMM^®^ [32]	SS	Sintering	Tubular	2–25	Filtration
Pall	PSS^®^ [100]	SS	Sintering	Tubular	5–55	Filtration
Pall	AccuSep™ [101]	SS, nickel-based alloys, ZrO_2_	Sintering or coating-sintering	Tubular	0.1–5	Microfiltration Gas and liquid filtration
Mott [76]	-	SS, nickel-based alloys, Ti	Sintering	Tubular	0.2–100	High temperature liquid or gas filtration for catalyst recovery Corrosive chemical filtration
Porvair Filtration	Sinterflo^®^ [29]	SS, nickel-based alloys, FeCrAl Alloy, Bronze	Sintering	Cylindrical	3–50	Filtration and separation for food and beverage, water treatment etc.
Graver Technologies, LLC	Scepter^®^ [102]	TiO_2_/SS	Coating–sintering	Tubular	0.1 or 0.02	Filtration (MF, UF)
Hyflux	FerroCep^®^ [103]	TiO_2_/SS	Coating–sintering	Tubular	0.1 or 0.02	Filtration (Fermentation broth clarification, starch processing, emulsified oil wastewater treatment, juice and syrup clarification)
Metalmembranes [104]	-	Metal oxide/Ti or Al	Plasma electrolytic oxidation–electrochemical machining	Plate	0.01–0.15	Detection and diagnostic applications
Sterlitech	Sterlitech™ [105]	Ag	Sintering	Disc	0.2–5	Analytical laboratory (XRD, SEM) Industrial hygiene Liquid clarification or sterilisation
Advanced Material Solutions (AMS) [106]	-	Ti	Coating–sintering	Tubular	0.05–5	Cross-flow filtration (MF, UF)
AMS [106]	-	Ti	Coating–sintering	Flat sheet	0.05–20	Filtration
AMS	DuraSter^©^ [30]	SS, high nickel alloys	Coating–sintering	Tubular	-	Filtration (MF)
